# Direct and Indirect Costs of Dinitrogen Fixation in *Crocosphaera watsonii* WH8501 and Possible Implications for the Nitrogen Cycle

**DOI:** 10.3389/fmicb.2012.00236

**Published:** 2012-07-20

**Authors:** Tobias Großkopf, Julie LaRoche

**Affiliations:** ^1^Research Division 2: Marine Biogeochemistry, Helmholtz-Centre for Ocean Research KielGEOMAR, Kiel, Germany

**Keywords:** *Crocosphaera watsonii*, diazotrophic niches, dinitrogen fixation, marine heterotrophic diazotrophy, nitrate, nitrogen cycle, oxygen minimum zone, underestimation of N_2_ fixation

## Abstract

The recent detection of heterotrophic nitrogen (N_2_) fixation in deep waters of the southern Californian and Peruvian OMZ questions our current understanding of marine N_2_ fixation as a process confined to oligotrophic surface waters of the oceans. In experiments with *Crocosphaera watsonii* WH8501, a marine unicellular diazotrophic (N_2_ fixing) cyanobacterium, we demonstrated that the presence of high nitrate concentrations (up to 800 μM) had no inhibitory effect on growth and N_2_ fixation over a period of 2 weeks. In contrast, the environmental oxygen concentration significantly influenced rates of N_2_ fixation and respiration, as well as carbon and nitrogen cellular content of *C. watsonii* over a 24-h period. Cells grown under lowered oxygen atmosphere (5%) had a higher nitrogenase activity and respired less carbon during the dark cycle than under normal oxygen atmosphere (20%). Respiratory oxygen drawdown during the dark period could be fully explained (104%) by energetic needs due to basal metabolism and N_2_ fixation at low oxygen, while at normal oxygen these two processes could only account for 40% of the measured respiration rate. Our results revealed that under normal oxygen concentration most of the energetic costs during N_2_ fixation (∼60%) are not derived from the process of N_2_ fixation *per se* but rather from the indirect costs incurred for the removal of intracellular oxygen or by the reversal of oxidative damage (e.g., nitrogenase *de novo* synthesis). Theoretical calculations suggest a slight energetic advantage of N_2_ fixation relative to assimilatory nitrate uptake, when oxygen supply is in balance with the oxygen requirement for cellular respiration (i.e., energy generation for basal metabolism and N_2_ fixation). Taken together our results imply the existence of a niche for diazotrophic organisms inside oxygen minimum zones, which are predicted to further expand in the future ocean.

## Introduction

The classical view on marine N_2_ fixation assumes that the preferred ecological niche of diazotrophs is largely limited to surface waters of oligotrophic areas, characteristically depleted of fixed N, saturated in dissolved oxygen and subjected to high light intensities (Howarth et al., 1988; Karl et al., 2002). However, recent surveys of the phylogenetic diversity and distributions of *nifH*, the functional gene marker for diazotrophy, demonstrated the presence of diazotrophs throughout all marine environments, ranging from deep-sea vents to highly productive shelf areas (Braun et al., 1999; Steppe and Paerl, 2002; Mehta et al., 2003; Zehr et al., 2003; Church et al., 2005; Langlois et al., 2005; Farnelid et al., 2011; Fernandez et al., 2011; Hamersley et al., 2011). Furthermore, in the euphotic zone of the Atlantic and Pacific oceans N_2_ fixation has been reported at high concentrations of ambient nitrate (Voss et al., 2004; Moisander et al., 2010; Sohm et al., 2011). Despite the broad diversity and distribution of diazotrophs, current research on oceanic N_2_ fixation focuses mainly on a few groups of cyanobacteria inhabiting the mixed layer, mainly the upper 200 m of the water column (Zehr et al., 2001, 2008; Voss et al., 2004; Staal et al., 2007a; Langlois et al., 2008; Moisander et al., 2010; Monteiro et al., 2010).

The high energetic costs associated with the process of N_2_ fixation has led to the general expectation that diazotrophs will be outcompeted by other microorganisms when nitrate is available. However, the costs of N_2_ fixation have mainly three origins, which need to be treated separately in order to understand why diazotrophs are poor competitors in eutrophic surface ocean areas. There are the costs of daily synthesis and degradation of the nitrogenase enzyme (Sherman et al., 1998; Taniuchi and Ohki, 2007; Mohr et al., 2010), there are the direct costs, in form of ATP and low potential electrons, to fuel the actual N_2_ fixation reaction (Eq. 1) and there are the indirect costs due to the removal of oxygen from the cellular interior during the period of N_2_ fixation.

Nitrogenase is highly sensitive to oxygen and is irreversibly inactivated on contact with molecular oxygen (Robson and Postgate, 1980; Gallon and Hamadi, 1984; Fay, 1992; Karl et al., 2002). In well oxygenated waters, such as the euphotic zone of oligotrophic oceans, diazotrophs must overcome the problem of removing dissolved oxygen from the immediate surroundings of the nitrogenase enzyme, at least during the period of active N_2_ fixation (Robson and Postgate, 1980; Gallon and Hamadi, 1984; Fay, 1992). For diazotrophic cyanobacteria not only the oxygen in the surrounding water becomes a problem, but also the oxygen generated by photosynthesis, since so far all cyanobacteria, with the exception of UCYN-A carry out oxygen-evolving photosynthesis (Bergman et al., 1997; Berman-Frank et al., 2003; Zehr et al., 2008; Tripp et al., 2010). Several strategies are employed by cyanobacteria to avoid oxidative damage during N_2_ fixation and all can be summarized as temporal or spatial separation of N_2_ fixation and photosynthetic oxygen evolution (Haselkorn, 1978; Gallon, 1981; Mitsui et al., 1986; Berman-Frank et al., 2001). Heterocystous filamentous cyanobacteria have developed specialized cells, called heterocysts where N_2_ fixation takes place. These cells lack oxygenic photosystem II and have a thick glycolipid layer that decreases diffusion of oxygen into the cell. They represent the classical example of spatial separation of N_2_ fixation and photosynthesis in an aerobic environment. Heterocysts rely on reduced carbon compounds from neighboring cells as an energy source and donate amino acids in exchange (Haselkorn, 1978). The costs for the removal of oxygen in heterocystous cyanobacteria are represented by the excess energy invested into producing a thick glycolipid layer to envelop the heterocysts. The unicellular diazotrophic cyanobacteria, which carry out photosynthesis and N_2_ fixation within the same cell, are at the other end of the spectrum. Most have developed a strategy of temporal separation, with oxygenic photosynthesis and carbon fixation during the light period and N_2_ fixation during the dark period when no oxygen evolution takes place (Mitsui et al., 1986; Sherman et al., 1998; Stoeckel et al., 2008; Toepel et al., 2008; Mohr et al., 2010; Shi et al., 2010). There are two theories of how unicellular diazotrophs overcome damage by oxygen: A strategy termed “respiratory protection,” consisting of increased respiration rates during the dark period, insures the removal of residual intracellular oxygen, thereby providing an anoxic environment for the nitrogenase enzyme to function properly (Peschek et al., 1991; Bergman et al., 1993). In the diazotrophic proteobacterium *Azotobacter vinelandii* an uncoupled cytochrome *bd* oxidase, that lacks the proton pumping activity and hence does not participate in energy production is active at elevated oxygen pressures (Poole and Hill, 1997). The alternative route for respiratory electrons toward a high affinity, low energy conserving oxidase acts like a relief valve during N_2_ fixation at high oxygen concentrations (Robson and Postgate, 1980). In cyanobacteria, genetic evidence points toward branched respiratory chains, with the possible involvement of uncoupled terminal oxidases like those in *Azotobacter*, but so far their activity has not been observed *in vivo* (Peschek et al., 1991, 2004; Hart et al., 2005; Paumann et al., 2005). The second strategy, termed autoprotection, implies that oxygen is permitted to diffuse into the cell but is removed by reduction through the nitrogenase enzyme itself, therefore competing with respiration and N_2_ fixation for electrons derived from storage carbohydrates (Oelze, 2000).

Both the respiratory protection and autoprotection mechanisms use electrons to reduce oxygen, either at the cell membrane or by the enzyme itself. Regardless of which strategy may be used by unicellular diazotrophic cyanobacteria, they present good model organisms to monitor the costs of N_2_ fixation, since the electrons used to reduce oxygen to water stem from reduced carbohydrates, buildup through photosynthesis during the daily light phase and thus can be monitored by the cellular carbon quota. We conducted laboratory experiments exposing cultures of *C. watsonii* WH8501, a unicellular diazotrophic cyanobacterium of ∼2.5–3 μm in diameter, to normal (20%) and low (5%) oxygen concentrations, measuring key physiological parameters over a 24-h period to establish an energetic budget of N_2_ fixing cells. Further, we calculated the direct costs of N_2_ fixation and compared them with the costs of ${\text{NO}}_{\text{3}}^{-}$ assimilation, the major competing nitrogen uptake process in the marine environment. We used the model organism *C. watsonii* to differentiate between the direct costs of N_2_ fixation, arising from the enzymatic reaction and the indirect costs arising from the combined removal of oxygen from the cellular interior and the repair of the nitrogenase due to oxidative damage. We found that this differentiation of the costs associated with N_2_ fixation is crucial when trying to understand how diazotrophs will compete along a vertical gradient in the ocean, when oxygen concentrations decrease but at the same time the availability of nitrate increases.

## Materials and Methods

### Culturing

All cultures of *Crocosphaera watsonii* WH8501 (Waterbury and Willey, 1988) were grown in YBCII Medium (Chen et al., 1996) at 28°C and 150 μmol Photons m^−2^ s^−1^ white illumination on a 12/12 light/dark cycle. Cultures where maintained in exponential growth phase and adapted to experimental conditions 8 days prior to the experiment. Subculturing was done in the exponential growth phase and 1/10 of the stock was used as inoculum. Cultures were held in 1 l Schott Duran glass bottles with magnetic stirrers at medium stirring speed to prevent sedimentation of cells during the experiment and bubbled with sterile filtered air (186 μM O_2_ in medium, referred to as normal oxygen) or with a 94.962% N_2_, 5% oxygen, and 0.038% CO_2_ mixture (46 μM O_2_ in medium, referred to as low oxygen; BASI-GASE). Daily cell counts where performed in a counting chamber (Neubauer improved) under the microscope. For the experiments, triplicate cultures were incubated under one given experimental condition (low or normal oxygen) and all parameters were measured every 3 h during a 24-h day cycle (L1, L2, L3, L4 are 0, 3, 6, and 9 h after the beginning of the light phase. L5/D1 is the beginning of the dark phase and D2, D3, D4, and D5 are 3, 6, 9, and 12 h into the dark phase, respectively). Linear regression analysis for growth rates of the nitrate experiment was performed with the statistics package of SigmaPlot (SYSTAT SOFTWARE).

### Oxygen measurements

Measurements of oxygen consumption and production were done in triplicates with an Oxytherm (Hansatech) Clark-type electrode unit with temperature stabilizer. Aliquots of 25 ml culture were centrifuged in a Beckman Avanti J-25 centrifuge at 4000 × *g* for 6 min. The pellet was resuspended in 2.5 ml of fresh YBCII medium. Two milliliters of this concentrated culture were placed in the cuvette of the oxygen electrode and the oxygen level was set to the approximate incubation level by gently bubbling with N_2_ gas or compressed air. After 10 min of initial dark adjustment the light was turned on three times at 150 μmol Photons m^**−**2^ s^**−**1^ for 5 min with 5 min dark intervals in between. Since the oxygen evolution and consumption rates showed an adaptive feature after switching on the light that lasted for 1–2 min, rates for photosynthesis and respiration were calculated from the last 3 min of the light and dark phases respectively, using the Oxygraph software (Hansatech) and normalized to cell numbers.

### Acetylene reduction measurements

Duplicate 2 ml culture aliquots were pipetted into 8.5 ml glass vials and sealed gas tight with a crimp cap containing a butyl rubber septum. Two vials containing sterile YBCII medium served as controls. The vials and medium were flushed with the appropriate gas mixture (5 or 20% Oxygen) for 60 s injected through a syringe needle. A second syringe needle served to release the pressure from the vial. Next, 650 μl acetylene were added to all vials with a gastight syringe (HAMILTON). Samples and controls were then incubated for 2 h at the corresponding temperature and light regime on a shaker (Wt 17; BIOMETRA). After the incubation period 250 μl of headspace gas from each vial were injected into a gas chromatograph (SHIMADZU GC-14B) equipped with a flame ionization detector. The area of the ethylene peak was converted to ppm with a calibration curve obtained by injecting 250 μl of 1, 10, 100, and 1000 ppm pure ethylene standards (Capone, 1993; Breitbarth et al., 2004).

### Particulate organic carbon/particulate organic nitrogen

Duplicates of 20 ml culture were filtered onto precombusted (12 h, 450°C) GF/F filters (WHATMAN). Filters were frozen and stored at **−**20°C until measurement. Blank filters served as control. Before measuring the nitrogen and carbon content, filters were placed over fuming HCl for 8 h and left to dry overnight at 60°C to remove remaining liquids and inorganic carbon. Filters were folded, rolled and packed tightly in a tin cup which was then combusted in a gas chromatograph (Elemental Analyzer, EUROVECTOR).

### Statistical analysis

Statistical analysis was performed with the STATISTICA software package (StatSoft). Error bars depicted in the figures represent standard deviations.

## Results

### Acetylene reduction, nitrogen fixation, and growth

**Figure 1:**
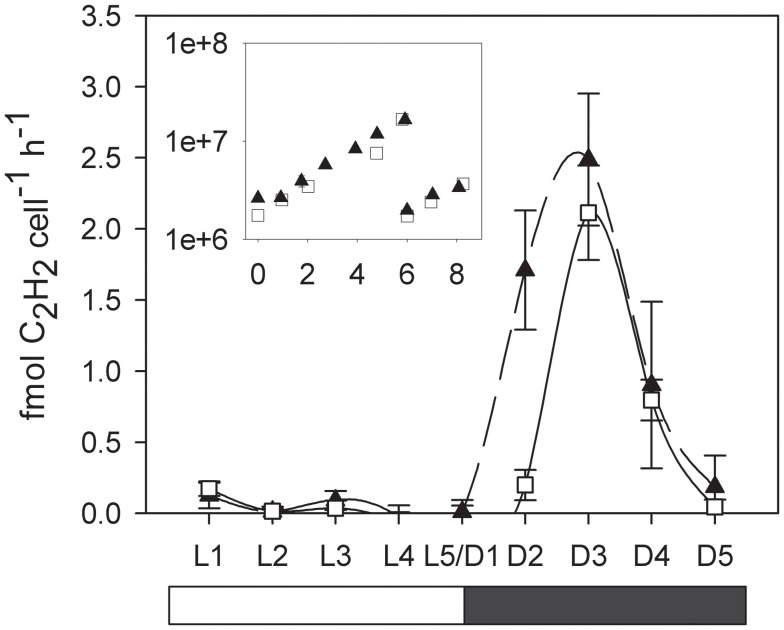
**Cellular acetylene reduction (AR) activity over a period of 24 h**. White squares and solid line, normal (20%) oxygen cultures. Black triangles and dashed line, low (5%) oxygen cultures. L1–L5 light phase, D1–D5 dark phase. Error bars denote standard deviations of triplicate cultures. Inlay: Cell numbers (*y*-axis: cells × ml^**−**1^, *x*-axis: days). Cultures where diluted with fresh medium on day 6 and the day cycle measurements were performed on day 8.

**Figure 2:**
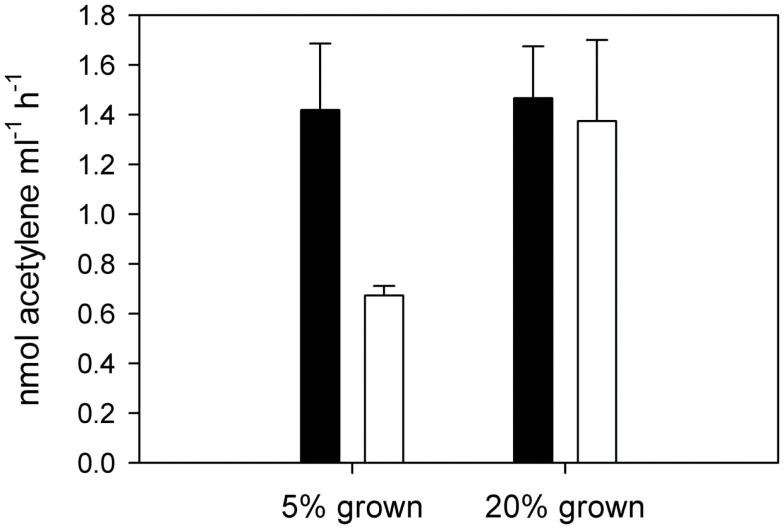
**Acetylene reduction by cultures of *Crocosphaera watsonii* grown under 5% oxygen (left) and 20% oxygen (right)**. Black bars represent AR measurements made under 5% oxygen atmosphere, white bars represent measurements made under 20% oxygen atmosphere. Error bars denote standard deviation of triplicate cultures.

The exponential growth rates established by cell counts during the course of the experiment were 0.28 ± 0.05 and 0.28 ± 0.02 for the low and normal oxygen treatments respectively. Although no differences in growth rates were observed between the low and normal oxygen treatments (independent *t*-test, df = 10, *p* = 0.88), other biochemical pathways showed distinct activity patterns during the diel cycle as a function of oxygen concentration. In cultures grown under a 12/12 light/dark cycle, acetylene reduction (AR) activity in *C. watsonii* began in the early dark phase, peaked around the middle of the dark phase and returned below the detection limit in the early light phase. At normal oxygen levels, peak rates of AR were reached 6 h after the beginning of the dark phase (D3) with 2.14 ± 0.34 fmol C_2_H_2_ cell^**−**1^ h^**−**1^. Under low oxygen conditions, peak rates were slightly but not significantly higher (2.48 ± 0.46 fmol C_2_H_2_ cell^**−**1^ h^**−**1^, independent *t*-test, df = 4, *p* = 0.34) but the AR activity started around 3 h earlier (D2; Figure 1). When cells grown at low oxygen were exposed to normal oxygen concentrations at the middle of the night phase (D3) they showed decreased AR rates, while cells acclimated to normal oxygen but bubbled with low oxygen immediately prior to AR rate measurements showed no change in their AR rate (Figure 2). This indicates that the nitrogenase activity is not energy limited due to a shortage in respiratory ATP production down to at least 5% ambient oxygen concentration. On the other hand a short-term increase in oxygen above the acclimated level led to an inhibition of the nitrogenase activity, possibly via the competition for electrons with respiration or the direct damage and inactivation of the enzyme from the contact with oxygen.

### Oxygen evolution and consumption

**Figure 3:**
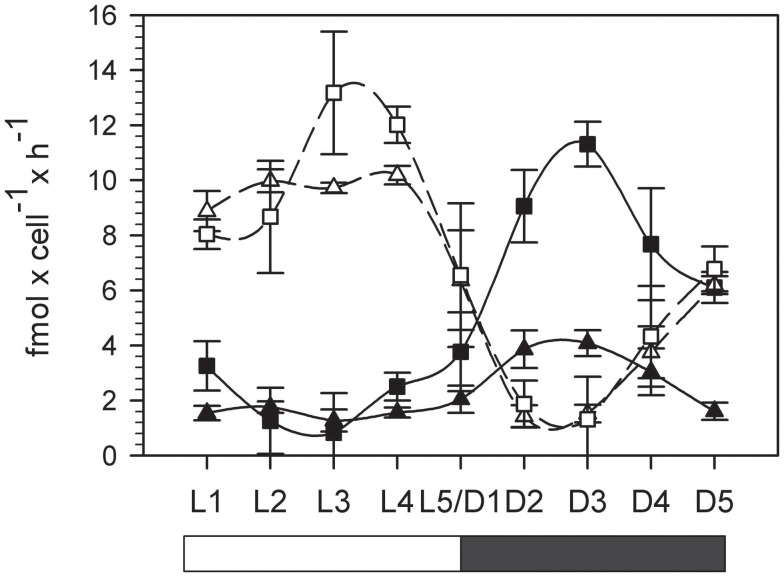
**Photosynthesis and oxygen consumption over a period of 24 h**. Squares: normal (20%) oxygen culture. Triangles: low (5%) oxygen culture. Filled symbols represent oxygen consumption in the dark, white symbols represent net oxygen evolution in the light (photosynthesis). L1–L5 light phase, D1–D5 dark phase. Error bars denote standard deviations of triplicate cultures.

The rates of net oxygen evolution (light phase) and consumption (dark phase) were integrated over the corresponding 12 h period and the results summarized in Table 1. Photosynthetic oxygen evolution peaked during the light phase and was higher (though not significantly) in the normal oxygen compared to low oxygen treatment (13.1 ± 2.3 and 10.2 ± 0.2 fmol cell^**−**1^ h^**−**1^ for normal and low oxygen respectively, independent *t*-test, df = 4, *p* = 0.07), however the 12-h integrated photosynthesis rate and total carbon buildup during the light phase were similar in both treatments (Figure 3; Table 1). The minimum in oxygen evolution was observed in both treatments in the middle of the dark phase (1.4 ± 0.4 and 1.3 ± 1.6 fmol cell^**−**1^ h^**−**1^ for normal and low oxygen respectively; Figure 3). The reduced capacity for oxygen evolution in the dark was paralleled by a decrease in Fv/Fm ratio that reached near zero values during the dark phase (data not shown). In contrast, the respiration rates peaked around 6 h after beginning of the dark phase (D3) and thus mirrored the AR activity. Under normal oxygen concentrations, the respiration rate in the dark increased by over a factor of 10 compared to background levels in the light phase (0.8 ± 1.4 fmol cell^**−**1^ h^**−**1^ at L3 and 11.3 ± 0.8 fmol cell^**−**1^ h^**−**1^ at D3, respectively). The respiration rates during the dark phase were significantly lower in the low oxygen growth conditions compared to the normal oxygen conditions (independent *t*-test, df = 4, *p* < 0.001) and only reached peak values of 4.1 ± 0.5 fmol cell^**−**1^ h^**−**1^, although the background respiration rates during the light phase were comparable to those of the normal oxygen grown cultures (1.3 ± 0.4 fmol cell^**−**1^ h^**−**1^ at L3, independent *t*-test, df = 4, *p* = 0.6; Figure 3).

**Table 1:** **Cellular budget of electron sources and sinks during the light (upper, white area) and the dark cycle (lower, gray area)**.

	5% Oxygen		20% Oxygen	
	fmol × 12 h^−1^ × cell^−1^	Electron equivalents (fmol)	fmol × 12 h^−1^ × cell^−1^	Electron equivalents (fmol)
Nett photosynthesis (O_2_ evolution)	98	392	102	408
Cellular carbon buildup (C)	96	−384	105	−420
Sum		8		−12
Respiration (O_2_ drawdown)	−38	−152	−102	−408
Nitrogen fixation (N buildup)	12	−36	11	−33
Cellular carbon breakdown (C)	−12	48	−77	308
Sum		−140		−133

*Oxygen and carbon are converted into electrons 4:1, nitrogen is converted 3:1*.

### Carbon and nitrogen content

**Figure 4:**
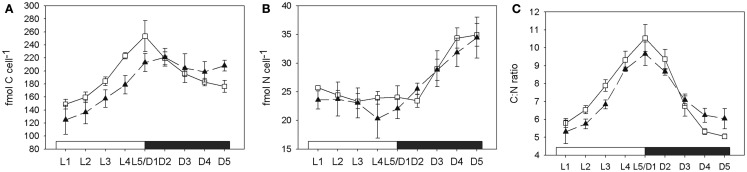
**Development of carbon (A), nitrogen (B) content in fmol per cell, and the C:N ratio (C) over the course of a 24-h cycle**. White squares and solid line, normal oxygen treatment. Filled triangles and dashed line, low oxygen treatment. L1–L5 light phase, D1–D5 dark phase. Error bars denote standard deviations of triplicate cultures.

The cellular carbon content increased during the light phase at comparable rates in both normal and low oxygen cultures. During the dark phase a significant decrease of cellular carbon content could only be measured in the culture grown under normal oxygen conditions, where about 30% of the cellular carbon levels at the end of the light phase were consumed again during the dark phase (generalized linear model (cellular carbon content decrease during the dark period). Low oxygen: *r*^2^ = 0.32, *p*(slope) = 0.3. Normal oxygen: *r*^2^ = 0.92, *p*(slope) < 0.01; Figure 4A). The cellular nitrogen content increased throughout the whole dark phase (D1–D5) in the low oxygen treatments, while at normal oxygen concentrations the cellular nitrogen content only increased between 3 and 9 h after the beginning of the dark phase (Figure 4B). At the end of the dark phase both treatments reached a cellular nitrogen level of around 150% of the level at the end of the light phase (L5/D1), thus fixing all nitrogen needed for a cell doubling in two consecutive nights. The molar C:N ratio in the low oxygen treatment decreased from the peak value of 9.7 ± 0.7 at D1 to 6 ± 0.6 at D5 over the course of the dark phase. In the normal oxygen treatment the decrease in the C:N ratio was more pronounced, due to the higher respiration and carbon drawdown rate. The value dropped from 10.5 ± 0.8 at D1 to 5.1 ± 0.1 at D5 (Figure 4C). In both the low and the normal oxygen treatment, the buildup of carbon during the day matched quite closely with the photosynthetic oxygen evolution integrated over the light cycle. However, the observed drawdown of carbon from D1 to D5 did not match the respiration rate integrated over the dark period. The difference between carbon drawdown and respiratory oxygen consumption had the same magnitude in both treatments, although the oxygen consumption and the cellular carbon drawdown differed by a factor of 2.7 and 6.4 between the treatments, respectively (Table 1). If carbon fixation continued during the dark, this would have masked the drawdown of storage carbohydrates. One enzyme that performs carbon fixation without consuming electrons is for example the phosphoenolpyruvate carboxylase (EC 4.1.1.31, GenBank accession: ZP_00517310) that produces oxaloacetic acid from phosphoenolpyruvate and CO_2_ consuming ATP. Oxaloacetic acid is an important precursor of many amino acids, so the phosphoenolpyruvate carboxylase could be used to produce carbon skeletons for N_2_ fixation in the dark. The electron imbalance could also arise from compounds in the cell other than carbon serving as electron donors or by a partial oxidation of carbon compounds without releasing them from the cell.

### Growth on nitrate

**Figure 5:**
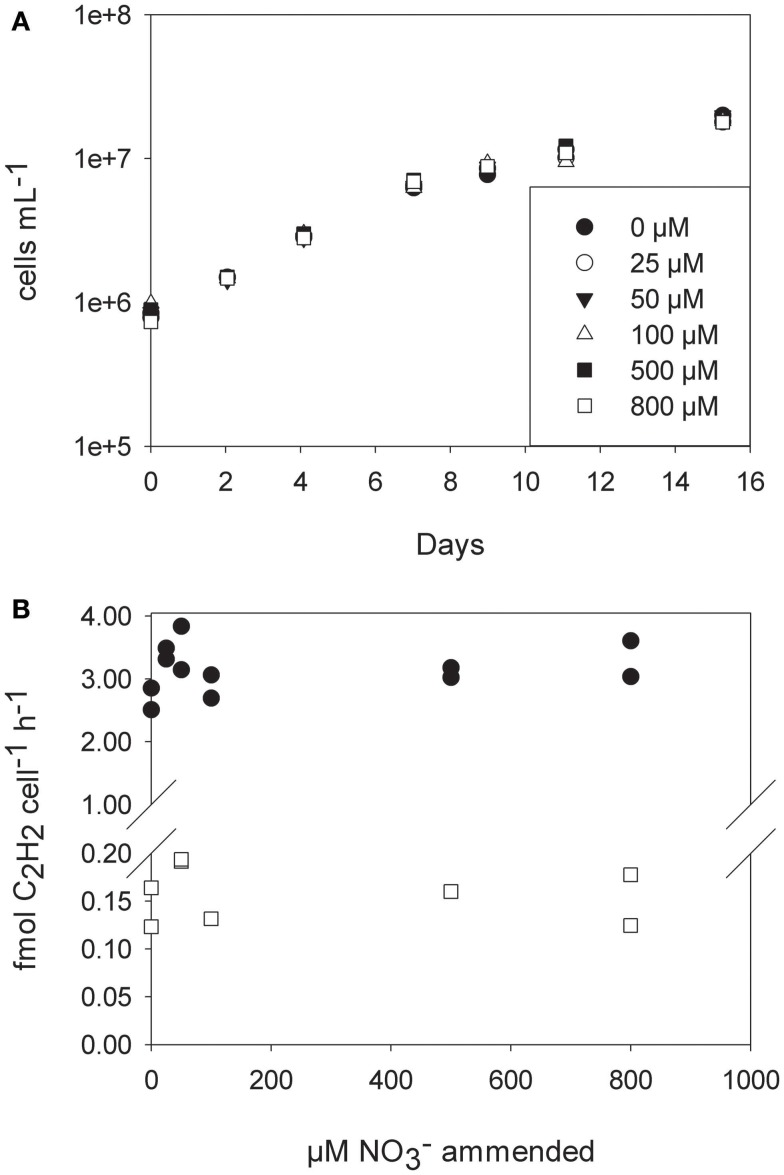
**(A)** Cell numbers (logarithmic scale) of *Crocosphaera watsonii* grown under different nitrate concentrations for 2 weeks. **(B)** Acetylene reduction assays were performed on day 4 (filled circles) and day 11 (open squares). Shown are duplicate measurements of acetylene reduction.

To test the growth rate of *C. watsnii* in the presence of nitrate, we grew batch cultures in YBCII medium amended with 25, 50, 100, 500, and 800 μM nitrate and an unamended control. There was no effect of nitrate concentration detectable on the growth rate (linear regression analysis of growth rate vs. nitrate concentration, slope not significantly different from 0, *p* = 0.68). Cell numbers increased at the same rate over the course of 14 days in all five treatments and the control. Nitrogenase activity measured by AR did not show a decrease under elevated levels of nitrate at day 4 after amendment with nitrate. Growth rates and AR rates decrease with time (e.g., day 11 vs. day 4), but trends were identical for all of the treatments (Figure 5).

### Costs of N_2_ fixation compared to assimilatory nitrate reduction

The net reaction for N_2_ fixation is

(1) $${\text{N}}_{\text{2}}+8{\text{H}}^{+}+8\text{F}{\text{d}}_{\text{red}}+16\text{ATP}=\text{2N}{\text{H}}_{\text{3}}+16\text{ADP}+\text{16}{\text{P}}_{\text{i}}+8\text{F}{\text{d}}_{\text{ox}}+{\text{H}}_{\text{2}}$$

**Figure 6:**
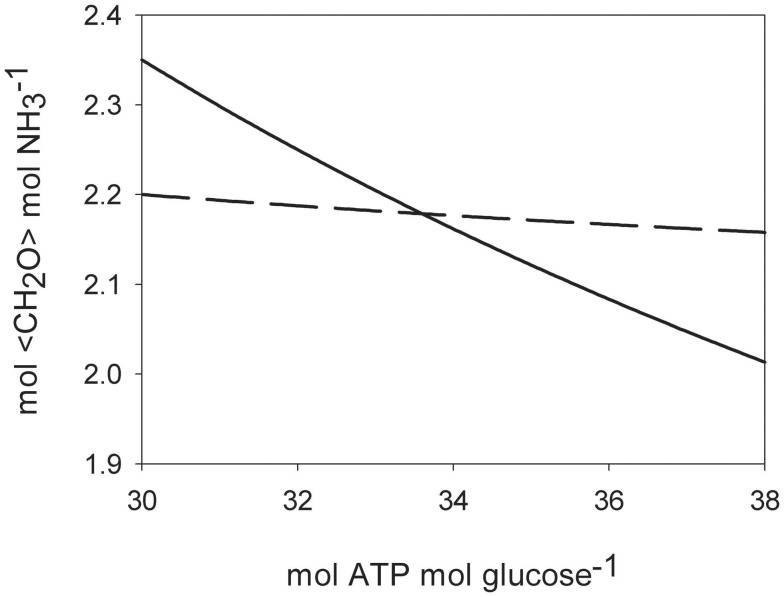
**Theoretical costs of ammonia assimilation in carbohydrate units (<CH_2_O>; mol mol^−1^) for different efficiencies of respiratory glucose oxidation**. Solid line: N_2_ fixation. Dashed line: assimilatory nitrate uptake.

With Fd_red_ and Fd_ox_, representing reduced and oxidized ferredoxin, respectively and P_i_, inorganic phosphate. It has been shown that diazotrophic cyanobacteria can recycle the two electrons from hydrogen with high efficiency through an uptake hydrogenase that uses ferredoxin as an electron acceptor (Robson and Postgate, 1980; Wilson et al., 2010), reducing Eq. 1 to 3 mol electrons (from reduced ferredoxin) and 8 mol ATP needed to convert half a mol of N_2_ into one mol of NH_3_. Assimilatory ${\text{NO}}_{\text{3}}^{-}$ reduction requires 8 mol electrons to reduce ${\text{NO}}_{\text{3}}^{-}$ to NH_3_ and one mol of ATP to get ${\text{NO}}_{\text{3}}^{-}$ into the cell per mol NH_3_ produced if an ATP-hydrolyzing ${\text{NO}}_{\text{3}}^{-}$ transporter is used (Herrero et al., 2001). Taking carbohydrates (e.g., glucose) as a common currency for both electrons and ATP sources, we can calculate the electrons and ATP requirements of both N_2_ fixation and assimilatory ${\text{NO}}_{\text{3}}^{-}$ reduction in terms of carbohydrate units. Table 2 summarizes the costs to produce one mol of NH_3_ using carbohydrate compounds as energy and electron donors. The calculations are made on the assumptions that one mole of glucose generates either 36 mol ATP via glycolysis and oxidative phosphorylation or 24 mol electrons. Some authors suggest a value of 30 mol ATP per mol glucose respired is reasonable (Raven, 2009), others quote 36 mol for mitochondria and heterotrophic bacteria (Martin and Muller, 1998). Figure 6 shows how the carbohydrate demand for N_2_ fixation and assimilatory nitrate reduction behaves over a range of theoretical ATP per glucose production rates from 30 to 38 (mol mol^**−**1^). Under the given conditions, the amount of energy generated by respiration is crucial in determining which N assimilation strategy is energetically more favorable, since it represents the converting agent of electrons into ATP. N_2_ fixation consumes more ATP, assimilatory nitrate reduction more low potential electrons. The higher the ATP yield per electron, the more favorable will the conditions be toward N_2_ fixation. The costs of N_2_ fixation are on a par with ${\text{NO}}_{\text{3}}^{-}$ assimilation at higher yields of ATP per mol glucose and could even be considered to show a slight advantage in terms of lower carbohydrate consumption per mol of NH_3_ assimilated, depending on the ATP production per mol of glucose respired (the efficiency of the respiratory energy conversion; Figure 6). In phototrophic organisms, the assimilatory reduction of nitrate is mediated by electrons coming directly from photosynthesis. These electrons on the other hand could be invested into carbon fixation, if not used for nitrate reduction. Therefore, in theory, it makes no difference, if the cell reduces CO_2_ to sugar with light energy and later uses the reduced carbon compounds to reduce nitrogen, or reduces nitrogen directly with light energy. In practice however, during every chemical conversion some of the energy is lost in form of heat. Therefore the indirect reduction of nitrogen via reduced carbohydrates should have a certain penalty attached. This specifically applies to unicellular phototrophic diazotrophs fixing N_2_ during the dark period, like *C. watsonii*. Other cyanobacteria, like the UCYN-A, capable of fixing N_2_ during the light period, could also directly use electrons from photosynthetic light reactions (Needoba et al., 2007; Tripp et al., 2010).

**Table 2:** **Costs of electrons and ATP needed to produce one mol of NH_3_ and the conversion into carbohydrate units**.

Energetic investment	Reduction of ^1^/_2_ N_2_ to ${\text{NH}}_{\text{4}}^{+}$	Reduction of ${\text{NO}}_{\text{3}}^{-}$ to ${\text{NH}}_{\text{4}}^{+}$
Mol electrons	3	8
Converted to mol <CH_2_O>	0.75	2.00
Mol ATP	8	1
Converted to mol <CH_2_O>	1.33	0.17
**Mol <CH_2_O> per ${\text{NH}}_{\text{4}}^{+}$ produced**	**2.08**	**2.17**

*It is assumed that 4 mol electrons or 6 ATP can be generated out of one mol of carbohydrates (<CH_2_O>, 1/6 mol of glucose). Bold represent the sum of electrons and ATP converted to glucose*.

### Energy utilization during N_2_ fixation

The moles of oxygen (O_2_) consumed per mol of nitrogen (N) fixed were 9.3:1 and 3.2:1 at normal and low oxygen respectively (Table 1). Using an ATP production of 4.28 mol ATP per mole of oxygen respired (Raven, 2009), the observed oxygen drawdown during the dark phase can be converted to 40 and 14 mol ATP per mol of nitrogen (N) fixed at normal and low oxygen, respectively. Table 1 sums up the cellular budget of oxygen, nitrogen, and carbon during the light and dark cycle for low and normal oxygen. According to Eq. 1, 8 mol ATP are needed for the direct reduction of 0.5 mol N_2_ to ammonium, so the nitrogenase enzyme reaction consumes 20 and 57% of the cellular energy produced in the dark at normal and low oxygen respectively. If a respiration rate of 1.5 fmol O_2_ cell^**−**1^ h^**−**1^ is assumed to meet the energetic demands of the basal metabolism (∼ background respiration at L3), 17.6 and 47% of the oxygen consumption during the dark phase can be accounted for at normal and low oxygen, respectively. Therefore, at low oxygen conditions the observed oxygen drawdown matches the calculated expenses of the cell (104%), whereas about 60% of the observed oxygen consumption at normal oxygen conditions appears in excess of the combined needs for N_2_ fixation and the basal metabolism. We assumed that the excess respiration represents the percentage of respiration invested into protection of the nitrogenase enzyme, either in the form of intracellular oxygen removal or energy necessary to repair the nitrogenase enzyme after oxidative damage.

## Discussion

The fact that diazotrophs need to protect the nitrogenase against high oxygen concentrations is well-known, consequently diazotrophs should thrive at low oxygen levels, such as those often found in the subsurface layers of the ocean (Robson and Postgate, 1980; Fay, 1992; Staal et al., 2007b; Compaore and Stal, 2010a). However, deeper layers of the ocean often hold substantially higher nitrate concentrations, presenting an N source alternative to N_2_ fixation. Although the direct energetic costs of N_2_ reduction to ammonium via nitrogenase are explicit and remain constant regardless of the environmental conditions, the costs associated with oxygen removal in diazotrophic cyanobacteria will vary with the dissolved oxygen concentration present in their habitat. Our results indicate that oxygen removal must be by far the largest energy sink in the daily life of a unicellular cyanobacterial diazotroph inhabiting fully oxygenated surface waters. The electrons from reduced carbon compounds serve a double function: They are used to reduce N_2_ to ammonium by the nitrogenase enzyme and they are channeled through the respiratory chain to generate energy in form of ATP. Thereby oxygen, the final electron acceptor in respiration, gets reduced to water. However, if the diffusion of oxygen into a *C. watsonii* cell is in excess of the respiratory demand, respiration has to increase proportionally to the oxygen concentration of the environment to prevent oxidative damage of the nitrogenase enzymes thereby creating an extra sink for electrons from storage carbohydrates. This generates a futile cycle consisting of an excess synthesis of carbohydrates during the light period needed to provide the reducing potential during the dark period to remove oxygen and protect nitrogenase. Very low oxygen concentrations will inhibit N_2_ fixation due to energy limitation (shortage of ATP production), while high oxygen concentrations necessitate an investment of extra energy into a protective mechanism. Between the two extremes is a narrow optimum, where oxygen is supplied at a concentration that meets, but does not exceed, the cellular energy demands thus circumventing the need to protect the nitrogenase against irreversible oxidative damage. This optimum will vary with the size and metabolic activity of the organism, with temperature, salinity, and energy supply (i.e., light regime in the case of *C. watsonii*), since all these parameters determine either the diffusion of oxygen into the cell or the oxygen demand by cellular respiration. During our experiment, the oxygen concentration of 5% oxygen (∼50 μmol l^**−**1^) could be considered close to the optimum for *C. watsonii*, since 104% of the observed respiration could be accounted for by energetic needs of basal metabolism and N_2_ fixation. Compaore and Stal (2010b) measured an optimum oxygen concentration of 7.5 and 5% at 30 and 76 μmol Photons^**−**1^ s^**−**1^ illumination for N_2_ fixation in *C. watsonii*, which is in good agreement with our findings. In the filamentous cyanobacteria *Nostoc* sp. and *Anabaena* the highest N_2_ fixation rates were obtained at 0% oxygen in the light, however the N_2_ fixation activity in the dark was highest around 5% oxygen (Compaore and Stal, 2010a). Similar results were obtained in *Trichodesmium*, where 0% oxygen produced highest N_2_ fixation rates at high light concentrations, while lower light intensities shifted the optimum toward 5% oxygen (Staal et al., 2007b).

Our results obtained with *C. watsonii* suggest that for diazotrophs inhabiting the oxygenated surface waters of the ocean the removal of oxygen at night to a level suitable for N_2_ fixation represents the largest expenditure in the energetic budget of the cells, exceeding the cost of the enzymatic reduction of N_2_ to ammonium. This extra energy expenditure to generate intracellular anaerobiosis in an aerobic environment likely contributes to the confinement of diazotrophs mostly to areas where fixed N compounds are scarce. The niche of surface ocean diazotrophs is therefore characterized by oligotrophy, or more specifically N-limitation. In these regions elevated costs associated with N-acquisition (N_2_ fixation) are not a disadvantage, simply because there is no competing option for obtaining fixed N available.

**Figure 7:**
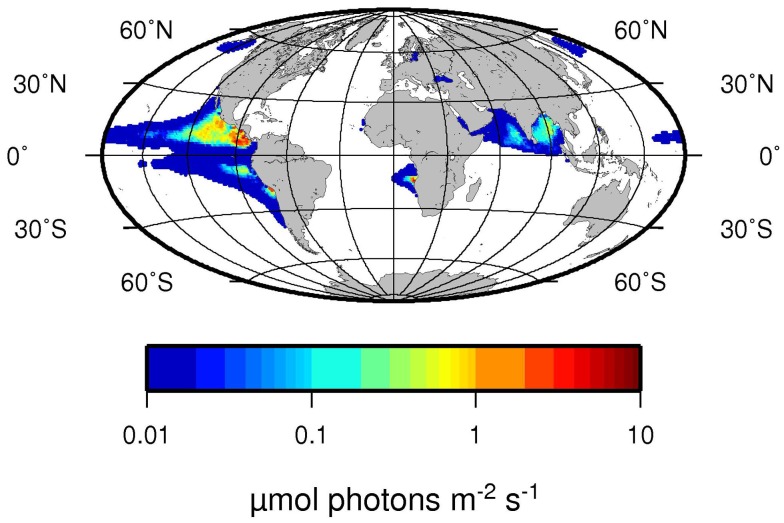
**Average light intensity at the depth of 25% oxygen saturation in μmol Photons m^−2^ s^−1^**. Note that 25% oxygen concentration corresponds to water equilibrated to a 5% oxygen containing atmosphere. Euphotic zone depth was derived from aquaMODIS satellite data of 2009 (http://oceancolor.gsfc.nasa.gov/) using the algorithm by Morel et al. (2007). Oxygen concentrations were taken from the World Ocean Atlas 2009 Garcia et al. (2010).

We propose here that a second niche possibly exists for unicellular diazotrophs, when N_2_ fixation reaches its energetic optimum at low oxygen concentrations. As a general pattern in the ocean, areas with low dissolved oxygen content are often high in dissolved nitrate, which would be considered unfavorable for N_2_ fixation. However, we could not detect any inhibitory effect of nitrate on the N_2_ fixation activity or any stimulating effect on the growth rates of *C. watsonii* up to a nitrate concentration of 800 μM. Similar results were obtained by others who found no inhibitory effect of nitrate on N_2_ fixation in *C. watsonii* up to 10 μM nitrate (Dekaezemacker and Bonnet, 2011). Therefore it seems that *C. watsonii* behaves indifferently toward nitrate and is neither stimulated nor inhibited by its presence, unlike the filamentous cyanobacteria *Trichodesmium* and *Anabaena*, that showed an inhibitory effect of nitrate additions on N_2_ fixation at 10 μM and 10 mM nitrate, respectively (Ramos and Guerrero, 1983; Mulholland et al., 2001). In a mixed community, such as those present in the ocean, an alternative mode of N_2_ fixation inhibition by nitrate would be the competitive disadvantage of N_2_-fixers against nitrate assimilating organisms, and the resulting competitive outgrowth (Agawin et al., 2007). However, we could show that the high energetic costs of N_2_ fixation and therefore most of the competitive disadvantage of N_2_ fixation against nitrate assimilation mostly results from the energetic costs of oxygen removal to generate an environment favorable for the fixation of N_2_. When omitting the additional costs arising from oxygen removal to protect the oxygen-labile nitrogenase, assimilatory ${\text{NO}}_{\text{3}}^{-}$ reduction and N_2_ fixation come in very close proximity in terms of energetic investment. Although several assumptions are necessary to calculate the energetic requirements, our calculations suggest that N_2_ fixation is as effective or slightly more effective then ${\text{NO}}_{\text{3}}^{-}$ uptake from a purely bioenergetics point of view, thus making N_2_ fixation a competitive lifestyle strategy even in the presence of high ${\text{NO}}_{\text{3}}^{-}$ concentrations. Hence, environments with low dissolved oxygen concentrations where diazotrophs do not need to expand extra energy for the removal of excess oxygen, can be considered optimal from the point of view of N_2_ fixation. The oxycline of an OMZ represents a gradient, where oxygen concentrations in some case range from fully saturated to zero or close to zero oxygen values. Along such a gradient, for any given diazotroph, there will be an oxygen concentration where the diffusion of oxygen into the cell meets the energetic demand of the diazotroph without creating an additional energy sink. Similar situations can be observed for rhizobia in a nodule of their host plant, in which the diazotroph will be supplied with just the right amount of oxygen necessary to meet the energetic demands of the N_2_ fixation reaction (Long, 1989). Unlike the situation for rhizobia, where the energy needed to reduce the oxygen concentration is supplied by the host plant (i.e., by the synthesis of leghemoglobins), an OMZ presents a situation where the oxygen concentration is reduced without any metabolic costs to the diazotroph or a possible symbiont. In the upper oxycline of the large OMZs, light availability can overlap with low oxygen and high nitrate concentrations (Figure 7), highlighting possible areas of where phototrophic diazotrophs could grow competitively. Although the extent of the areas in Figure 7 that would favor photosynthetic diazotrophs are currently limited, predicted future shoaling and expansion of the OMZs (Stramma et al., 2009) may also lead to an increase in this additional niche for diazotrophs. In addition to the oxycline of OMZs, such environments could develop at the surface of particles and aggregates where high community respiration rates may prevail (Paerl et al., 1995). While our results were obtained on unicellular photosynthetic cyanobacteria, the theoretical suggestions should be applicable to other diazotrophs as well. Recently, high rates of N_2_ fixation were detected in the OMZs of Peru and California, performed by diazotrophs other than cyanobacteria (Fernandez et al., 2011; Hamersley et al., 2011). Yet the N_2_ fixation activity of these organisms does not seem to be inhibited by the presence of high nitrate concentrations in the OMZs.

Furthermore, heterotrophs, living on energy sources low in ammonium compared to carbon (organic material with high C:N ratio) need extra N-sources to meet their nitrogen demand. Such organisms could assimilate nitrate or, if the oxygen concentration is lowered to an optimum level, fix N_2_ despite high nitrate concentrations. We would therefore expect heterotrophic diazotrophy in oxygen minimum zones to be mostly dependent on the C:N ratio of the energy supply, rather than on the DIN:DIP (dissolved inorganic nitrogen, dissolved inorganic phosphorous) ratio, like the diazotrophy of the surface oceans (Deutsch et al., 2007).

## Conflict of Interest Statement

The authors declare that the research was conducted in the absence of any commercial or financial relationships that could be construed as a potential conflict of interest.
